# Misidentification of *Candida guilliermondii* as *C. famata* among Strains Isolated from Blood Cultures by the VITEK 2 System

**DOI:** 10.1155/2014/250408

**Published:** 2014-05-29

**Authors:** Si Hyun Kim, Jeong Hwan Shin, Jeong Ha Mok, Shine Young Kim, Sae Am Song, Hye Ran Kim, Joong-Ki Kook, Young-Hyo Chang, Il Kwon Bae, Kwangha Lee

**Affiliations:** ^1^Department of Laboratory Medicine, Inje University College of Medicine, Busan 614-735, Republic of Korea; ^2^Paik Institute for Clinical Research, Inje University College of Medicine, Busan 614-735, Republic of Korea; ^3^Department of Internal Medicine, Pusan National University School of Medicine, Busan 602-739, Republic of Korea; ^4^Biomedical Research Institute, Pusan National University School of Medicine, Busan 602-739, Republic of Korea; ^5^Department of Laboratory Medicine, Pusan National University School of Medicine, Busan 602-739, Republic of Korea; ^6^Department of Oral Biochemistry, School of Dentistry, Chosun University, Gwangju 501-759, Republic of Korea; ^7^Korean Collection for Type Cultures, Biological Resource Center, KRIBB, Daejeon 305-806, Republic of Korea; ^8^Department of Dental Hygiene, College of Medical and Life Science, Silla University, Busan 617-736, Republic of Korea; ^9^Department of Internal Medicine, Pusan National University Hospital, 179, Gudeok-ro, Seo-gu, Busan 602-739, Republic of Korea

## Abstract

*Introduction*. The aim of this study was to differentiate between *Candida famata* and *Candida guilliermondii* correctly by using matrix-assisted laser desorption/ionization-time of flight mass spectrometry (MALDI-TOF MS) and gene sequencing. *Methods*. Twenty-eight *Candida* strains from blood cultures that had been identified as *C. famata* (*N* = 25), *C. famata*/*C. guilliermondii* (*N* = 2), and *C. guilliermondii* (*N* = 1) by the VITEK 2 system using the YST ID card were included. We identified these strains by MALDI-TOF MS and gene sequencing using the 28S rRNA and *ITS* genes and compared the results with those obtained by the VITEK 2 system. *Results*. All 28 isolates were finally identified as *C. guilliermondii.* Sequencing analysis of the 28S rRNA gene showed 99.80%–100% similarity with *C. guilliermondii* for all 28 strains. The *ITS* gene sequencing of the strains showed 98.34%–100% homology with *C. guilliermondii.* By MALDI-TOF, we could correctly identify 21 (75%) of 28 *C. guilliermondii* isolates. *Conclusion*. We should suspect misidentification when *C. famata* is reported by the VITEK 2 system, and we always should keep in mind the possibility of misidentification of any organism when an uncommon species is reported.

## 1. Introduction

Bloodstream infections caused by* Candida* species have increased significantly over recent decades and are associated with high rates of morbidity and mortality [1, 2]. A rapid and accurate identification of* Candida* species is of great importance to the selection of appropriate antifungal agents and for appropriate patient management [3].

We have faced an increase in* Candida famata* isolation from blood cultures with the use of the VITEK 2 system in the clinical laboratory. This organism usually is found on natural substrates and has been reported as a rare pathogen of human beings [4–7].* Candida famata* and* C. guilliermondii* are extremely difficult to differentiate by phenotypic features [8, 9], so we need to determine whether the recent increase of* C. famata* in the blood is true or reflects an error by the identification system because of the organism's similarity in biochemical characteristics to other* Candida* spp.

The aim of this study was to identify these strains correctly using matrix-assisted laser desorption/ionization-time of flight mass spectrometry (MALDI-TOF MS) and 28S rRNA and* ITS* gene sequencing.

## 2. Materials and Methods

### 2.1. Strains

Twenty-eight nonduplicated* Candida* strains identified as* C. famata* (*N* = 25),* C. famata/C. guilliermondii* (*N* = 2), or* Candida guilliermondii* (*N* = 1) by the VITEK 2 system using the YST-ID card were included. All 28 strains were collected from blood culture at Inje University Busan Paik Hospital in the Republic of Korea between January 2007 and December 2008. We selected 25 nonduplicated strains identified as* C. famata* (identification scores 92%–95%) and 2 nonduplicated strains showing a result of* C. famata*/*C. guilliermondii* (50%/50%) by the VITEK system. We selected these strains to achieve even distribution throughout the isolation period and the admission ward. One* Candida guilliermondii* isolate (97%) was used as a control. All isolates were stored in skim milk at −80°C until testing.

### 2.2. Phenotypic Characterization

Fungal culture and identification were performed by standard procedures in a clinical microbiology laboratory. Yeast-form fungi were identified according to conventional biochemical laboratory methods by the VITEK 2 system using YST-ID. All procedures were done according to the manufacturer's instruction. We repeated the identification procedures twice using the same YST-ID for all strains.

### 2.3. MALDI-TOF MS Analysis

We identified all strains with MALDI-TOF MS using MALDI Biotyper. MALDI-TOF analyzes the unique protein spectra produced by extracts of microbial cells. First, *α*-cyano-4-hydroxycinnamic acid (HCCA portioned, number 255344, Bruker Daltonik GmbH, Bremen, Germany) was prepared as the MALDI matrix for Bruker MALDI Biotyper measurements. Colonies were transferred to a steel target, namely, MSP 96 polished steel (Bruker Daltonics), and overlaid with 1 *μ*L of matrix solution directly after drying. The extraction steps were done as follows. Briefly, samples were prepared using formic acid and acetonitrile after alcohol treatment, and then 1 *μ*L of extract supernatant fluid was used for analysis. Spectra were automatically concentrated on a maximum of 240 shots by MBT autoX and then compared with the Bruker Daltonics database using the MALDI Biotyper RTC software. We repeated test with extraction method if no result was obtained.

### 2.4. Gene Sequencing

All* Candida* strains were identified by polymerase chain reaction (PCR) and by direct sequencing of the 28S rRNA and* ITS* genes. The fungal genomic DNA collected from a single colony of an overnight culture was extracted with InstaGene Matrix kit (Bio-Rad Laboratories, Hercules, CA USA) according to the manufacturer's recommendation.

The* ITS* gene was amplified using the universal fungal primers ITS1 (5′-TCC GTA GGT GAA CCT GCG G-3′) and ITS4 (5′-TCC TCC GCT TAT TGA TAT GC-3′). The 28S rRNA gene was amplified with the primers D1/D2-F (5′-GCA TAT CAA TAA GCG GAG GAA AAG-3′) and D1/D2-R (5′-GGT CCG TGT TTC AAG ACG G-3′) as previously described [10]. The primers for sequencing were the same as those for PCR amplification. Both strands of the purified DNA from the PCR were sequenced directly with a Big Dye Terminator Cycle Sequencing kit (Applied Biosystems, Foster City, CA) and the ABI PRISM 3130 genetic analyzer (Applied Biosystems). The sequences were compared with those of the type and reference strains to confirm species identification using NCBI (a genome database of the National Center for Biotechnology Information). Phylogenetic analyses were performed for the 28S rRNA and* ITS* using the neighbor-joining method with MEGA version 4.

## 3. Results

All 28 isolates were finally identified as* C. guilliermondii*, although 27 had been reported as* C. famata* (*N* = 25) or* C. famata*/*C. guilliermondii* (*N* = 2) by the VITEK 2 system using the YST-ID card. There was no true* C. famata* strain. Thus, we could confirm that* C. famata* is a rare cause of fungemia, its diagnosis being attributable to the misidentification of* C. guilliermondii* as* C. famata* by the VITEK 2 system.

The MALDI-TOF MS method was valuable for identification of* C. guilliermondii*. We could correctly identify 21* C. guilliermondii* isolates that had been identified as* C. famata* (*N* = 18),* C. famata/C. guilliermondii* (*N* = 2), or* C. guilliermondii* (*N* = 1) by VITEK 2. Two strains showed a score of more than 2.0 by the Bruker MALDI Biotyper, whereas the scores of 19 strains were between 1.7 and 1.99. The remaining seven isolates could not be identified to the species level even though we retested and used the extraction method. They showed scores of less than 1.7, and we defined the result as no identification (Table 1).

**Figure 1:**
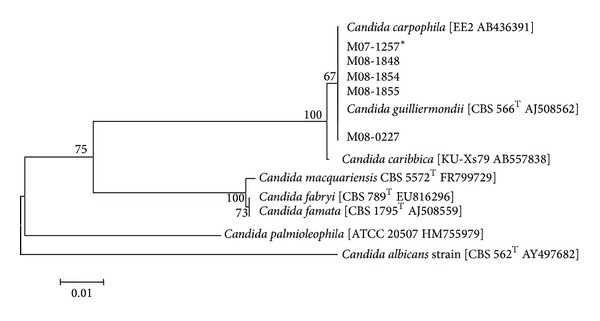
Phylogenetic analysis using the neighbor-joining method based on the 28S rDNA gene sequence for 28 clinical isolates and type and reference strains. The scale bar represents the distance between strains.*M07-1257 represents other strains that have 100% sequence similarity to purported 21* C. famata*, 1* C. guilliermondii*, and 2* C. famata*/*C. guilliermondii* strains.

**Figure 2:**
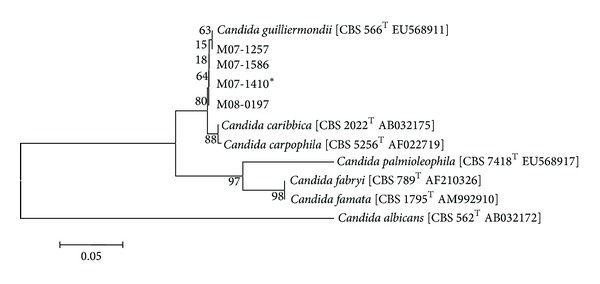
Phylogenetic analysis using the neighbor-joining method based on the* ITS* sequence for 28 clinical isolates and type and reference strains. The scale bar represents the distance between strains. *M07-1410 represents strains that have 100% sequence similarity to the purported 21* C. famata*, 1* C. guilliermondii*, and 2* C. famata*/*C. guilliermondii* strains.

The final correct identification could be acquired from the use of 28S rRNA and* ITS* sequencing. We compared the analyzed sequences from the clinical isolates with those of type and reference strains obtained from the NCBI database. By using 28S rRNA gene sequencing, all the 28 isolates were clearly identified as* C. guilliermondii* with a similarity between 99.80% and 100%, and these strains also showed close similarity to* C. carpophila* (99.65%–99.82%) and* C. caribbica* (99.30%–99.47%). For* ITS* sequencing, all 28 isolates were first identified as* C. guilliermondii*, showing similarity between 98.34% and 100%. However, the similarity with* C. caribbica* was also very high (99.03%–99.23%). To differentiate these closely related species, we constructed a phylogenetic tree using the neighbor-joining method with MEGA version 4. By this method, all strains were clustered with* C. guilliermondii*, and these were obviously distinguished from* C. famata* by both genes. For the phylogenetic tree of the 28S rRNA gene,* C. guilliermondii* strains were clearly differentiated from* C. caribbica*, but not from* C. carpophila* (Figure 1). When using the* ITS* sequence, all isolates were clustered as one group with* C. guilliermondii*, and this group was separated from* C. caribbica* and* C. carpophila* (Figure 2).

## 4. Discussion

*Candida* species are the fourth most common cause of nosocomial bloodstream infections [11].* Candida albicans* is still the most common species isolated from human beings; however, the frequency of non-*albicans Candida* species is increasing as a major cause of catheter-related bloodstream infections especially [12].

*Candida famata* is a rare cause of invasive infection. This strain was described as* Torula candida* after being discovered in Japan [13]. It then was called* Torulopsis famata* and* Debaryomyces hansenii* and finally defined as* C. famata*. It occupies the human skin, vagina, and oral cavity as a colonizing organism, so it has been considered a contaminant even though it has been isolated from clinical specimens [13, 14].

However, there are several reports concerning invasive candidiasis caused by* C. famata*, and it should be considered an important opportunistic pathogen. The most important disease caused by* C. famata* is intravenous catheter-associated candidemia in immunocompromised patients, as described by St.-Germain and Laverdiere [7] in a bone marrow transplant patient. It also has been detected in patients with endophthalmitis with chronic intraocular inflammation [15], candidemia with aplastic anemia, and central catheterization [13] and fatal peritonitis in a patient undergoing continuous ambulatory peritoneal dialysis [16].

In clinical laboratories, most bacteria and yeasts are routinely identified by biochemical characteristics using a commercial kit or automated identification system such as API and VITEK. Renneberg et al. [17] reported evaluation by the Staph ID 32 and StaphZym systems for coagulase-negative staphylococci showing a high rate of misidentification. This phenomenon is obvious when some species have high similarity in biochemical reactions. Misidentification also is possible for* Candida* species when commercial kits such as API 20C are used for identification [10].* Candida famata* is very similar in biochemical characteristics to* C. guilliermondii* and* C. caribbica*. Desnos-Ollivier et al. [9] reported that several* C. famata* strains identified by API 32C were* C. guilliermondii, C. haemulonii, C. lusitaniae*, and* C. palmioleophila* when gene sequencing was done. More recently, there was an interesting report of misidentification of* C. parapsilosis* as* C. famata* in vertebral osteomyelitis [18]. The authors of that paper asserted the importance of this problem because of differences in antifungal susceptibility [3], especially the fact that the resistance rate to fluconazole and amphotericin B is high in* C. guilliermondii*. Similar misidentification can be seen in the identification of mold-form fungi [19]. We agree with their recommendation concerning the importance of using many molecular techniques for diagnosis of infectious diseases to overcome the limits of conventional methods [20, 21].

The VITEK 2 system is one of the most common automated identification systems using a colorimetric identification card of YST. In previous reports, it was noted to be of high sensitivity and specificity greater than 95% for common* Candida* species isolated from clinical specimens compared with molecular methods [22, 23]. On the other hand, the identification rates for* C. parapsilosis* by the VITEK 2 system were reported to be as low as 71.7%, although it can be improved to 93.3% by examining the morphologic features on cornmeal agar plates [23].

At first, we reported the results as* C. famata* for 25 strains because the identification scores were high (identification scores: 92%–95%) by the VITEK 2 system. However, the isolation of* C. famata* by the VITEK 2 system has increased in our clinical microbiology laboratory, and this is unusual for us. So we assumed either the possibility of a change in the distribution of* Candida* species isolated from blood culture or some error in identification by the VITEK 2 system. We tried to identify these isolates correctly using MALDI-TOF MS and 28S rRNA and* ITS* gene sequencing analysis, and we finally confirmed the misidentification by the VITEK 2 System of* C. guilliermondii* as* C. famata* in clinical isolates from blood culture.

Many microbes show similar patterns of biochemical reactions, and we have difficulty in identification. Broad-range PCR and gene sequencing is a good tool for the correct identification of fungi, and 28S rRNA and* ITS* are well known as useful regions for identification of fungi [24]. This technique allows more accurate identification of* Candida* species based on differences in the rRNA [25, 26]. In this study, all isolates were identified as* C. guilliermondii* using the 28S rRNA and* ITS* genes. We found that the 28S rRNA gene could not discriminate between* C. guilliermondii* and* C. carpophila* because of the high similarity of their sequences. However, these regions could distinguish between* C. famata* and* C. guilliermondii*.

In recent years, MALDI-TOF MS was introduced as a technique for molecular identification [27]. This technique has a profound advantage of rapid identification to the species level within a few minutes. We evaluated its ability to correctly identify* C. guilliermondii*. We analyzed these 28* Candida* strains according to the recommendations of the manufacturers. Among the 28* Candida* species, two isolates showed a score of more than 2.0 and were* C. guilliermondii*. Nineteen isolates gave a result of* C. guilliermondii*, but the scores were between 1.7 and 2.0. We got nonreliable results for 7 strains even though we retested after extraction. Stevenson et al. [28] evaluated the clinical usefulness of MALDI-TOF MS for the identification of yeasts by their own library using 109 reference and type strains. They could identify isolates correctly to the species level in 192 (97.5%) of 197 isolates with 100% correct identification of all 15* C. guilliermondii*. Lacroix et al. [29] compared two MALDI-TOF MS systems, Andromas and Bruker MaldiBiotyper, with conventional identification methods using 1383 clinical* Candida* isolates. The correct identification rates of the two MALDI-TOF MS systems (98.2%) were higher than those of conventional methods (96.5%) by comparison with sequencing results. Bruker MaldiBiotyper recommends that a score greater than 2.0 be identified to the species level and to the genus level if the values are greater than 1.70 but lower than 2.0. It gives “Not reliable identification” if the values are lower than 1.70. However, only 2 strains were higher than 2.0 in this study, so we would recommend a cutoff value of 1.7 to identify* C. guilliermondii*.

In conclusion, we confirmed the misidentification of* C. guilliermondii* as* C. famata* by the VITEK 2 YST system. We now suspect misidentification when* C. famata* is reported by the VITEK 2 system, and we always keep in mind the possibility of misidentification when an uncommon species is reported.

## Figures and Tables

**Table 1:** VITEK 2 system, MALDI-TOF MS, and sequencing results for 28 *Candida* strains.

Strain	VITEK 2		MALDI-TOF MS		Sequencing			
	Identification	Score	Identification	Score	ID (28S gene)	Similarity	ID (*ITS* gene)	Similarity
M07-1257	*C. guilliermondii *	97%	*C. guilliermondii *	1.791	*C. guilliermondii *	100%	*C. guilliermondii *	100%
M07-1410	*C. famata *	95%	*C. guilliermondii *	1.782	*C. guilliermondii *	100%	*C. guilliermondii *	99.81%
M07-1525	*C. famata *	95%	*C. guilliermondii *	1.882	*C. guilliermondii *	100%	*C. guilliermondii *	99.81%
M07-1575	*C. famata *	95%	No ID*	1.682	*C. guilliermondii *	100%	*C. guilliermondii *	99.81%
M07-1586	*C. famata *	95%	*C. guilliermondii *	1.878	*C. guilliermondii *	100%	*C. guilliermondii *	98.34%
M07-1601	*C. famata *	93%	*C. guilliermondii *	1.951	*C. guilliermondii *	100%	*C. guilliermondii *	99.81%
M07-1627	*C. famata *	95%	*C. guilliermondii *	1.864	*C. guilliermondii *	100%	*C. guilliermondii *	99.81%
M07-1639	*C. famata *	95%	No ID*	1.700	*C. guilliermondii *	100%	*C. guilliermondii *	99.81%
M08-0109	*C. famata *	95%	*C. guilliermondii *	1.832	*C. guilliermondii *	100%	*C. guilliermondii *	99.81%
M08-0121	*C. famata *	95%	*C. guilliermondii *	1.951	*C. guilliermondii *	100%	*C. guilliermondii *	99.81%
M08-0160	*C. famata *	95%	*C. guilliermondii *	1.917	*C. guilliermondii *	100%	*C. guilliermondii *	99.81%
M08-0197	*C. famata *	95%	*C. guilliermondii *	1.719	*C. guilliermondii *	100%	*C. guilliermondii *	99.63%
M08-0217	*C. famata *	95%	*C. guilliermondii *	1.892	*C. guilliermondii *	100%	*C. guilliermondii *	99.81%
M08-0227	*C. famata *	95%	*C. guilliermondii *	1.726	*C. guilliermondii *	99.80%	*C. guilliermondii *	99.81%
M08-0296	*C. famata *	95%	*C. guilliermondii *	1.872	*C. guilliermondii *	100%	*C. guilliermondii *	99.81%
M08-0328	*C. famata/C. guilliermondii *	50%/50%	*C. guilliermondii *	2.068	*C. guilliermondii *	100%	*C. guilliermondii *	99.81%
M08-1839	*C. famata *	92%	No ID*	1.615	*C. guilliermondii *	100%	*C. guilliermondii *	99.81%
M08-1847	*C. famata *	95%	No ID*	1.492	*C. guilliermondii *	100%	*C. guilliermondii *	99.81%
M08-1848	*C. famata *	95%	*C. guilliermondii *	1.825	*C. guilliermondii *	99.80%	*C. guilliermondii *	99.81%
M08-1849	*C. famata *	95%	No ID*	1.394	*C. guilliermondii *	100%	*C. guilliermondii *	99.81%
M08-1850	*C. famata *	95%	*C. guilliermondii *	2.030	*C. guilliermondii *	100%	*C. guilliermondii *	99.81%
M08-1851*	*C. famata/C. guilliermondii *	50%/50%	*C. guilliermondii *	1.992	*C. guilliermondii *	100%	*C. guilliermondii *	99.81%
M08-1852	*C. famata *	95%	No ID*	1.649	*C. guilliermondii *	100%	*C. guilliermondii *	99.81%
M08-1854	*C. famata *	95%	*C. guilliermondii *	1.883	*C. guilliermondii *	99.80%	*C. guilliermondii *	99.81%
M08-1855	*C. famata *	95%	No ID*	1.637	*C. guilliermondii *	99.80%	*C. guilliermondii *	99.81%
M08-1857	*C. famata *	95%	*C. guilliermondii *	1.854	*C. guilliermondii *	100%	*C. guilliermondii *	99.81%
M08-1876	*C. famata *	95%	*C. guilliermondii *	1.722	*C. guilliermondii *	100%	*C. guilliermondii *	99.81%
M08-1898	*C. famata *	92%	*C. guilliermondii *	1.939	*C. guilliermondii *	100%	*C. guilliermondii *	99.81%

*No ID: not reliable identification.
